# Biological functions and molecular mechanisms of exosome-derived circular RNAs and their clinical implications in digestive malignancies: the vintage in the bottle

**DOI:** 10.1080/07853890.2024.2420861

**Published:** 2024-11-01

**Authors:** Yuanyan Lou, Jianing Yan, Qingqing Liu, Min Miao, Yongfu Shao

**Affiliations:** a Department of Gastroenterology, the First Affiliated Hospital of Ningbo University, Ningbo, China; b Health Science Center, Ningbo University, Ningbo, China

**Keywords:** Exosomal circRNA, exosomes, digestive malignancies, biological function, molecular mechanism, clinical implication

## Abstract

**Background:**

Circular RNAs (circRNAs) are identified as a novel family of endogenous RNA molecules through ‘back-splicing’ and covalently linked at the 5′ and 3′ ends. Emerging researches have demonstrated circRNAs are stable and abundant in exosomes called exosomal circRNAs (exo-circRNA).

**Materials and methods:**

We searched recent studies and references to summary the research progress of exosomal circRNA.

**Results:**

Recent studies have revealed that exosome-derived circRNAs including exo-CDR1as, exo-circRanGAP1, exo-circIAR play vital roles in cell proliferation and apoptosis, epithelial mesenchymal transition, invasion and metastasis, angiogenesis, immune evasion, cellular crosstalk, cancer cachexia through a variety of biological mechanisms, such as serving as microRNA sponges, interacting with RNA binding proteins, regulating gene transcription, N6-Methyladenosine modification and so on. Due to their characteristics of origin, structure, properties and biological functions, exo-circRNAs are expected to apply in precious diagnosis and prognostic indicators, improving drug and radiation resistance and sensitivity, becoming biological therapeutic targets.

**Conclusion:**

We summarize the update of digestive malignancies associated exo-circRNAs in biogenesis, biological functions, molecular mechanisms, clinical implications, potential applications and experimental technique in order to effectively promote transformation and application in the future.

## 1. Introduction

Digestive malignancies seriously threaten human life. With the characteristics of high incidence and mortality, digestive malignancies have become the one of the highest incidence of cancer diseases in different human body system [1]. Due to complex pathogenesis, difficulties in early diagnosis and easy metastasis of digestive malignancies, the prognosis of most patients is still unsatisfactory though clinical screening methods and targeted therapy are currently strengthened [2]. More biomolecular and related transformation researches are needed to improve the current clinical situation.

Exosomes are a class of encapsulated nanoscale vesicular solid structures, composed of lipid bilayer vesicular membranes, which contain circular RNA (circRNA), microRNA (miRNA), proteins, and so on [3]. Exosomes act as a ‘messenger’ between cells, involving the process of tumorigenesis, evolution, metastasis and immune escape [3]. Circular RNAs (circRNAs) are a class of novel endogenous RNA molecules through ‘back-splicing’ and covalently linked at the 5′ and 3′ ends [4]. Emerging researches have demonstrated circRNAs are stable and abundant in exosomes called exosomal circRNAs (exo-circRNA) [5]. Exo-circRNAs are one of the most important information materials in exosomes. Recent studies have revealed that exosome-derived circRNAs, in the form of exosomes, involve in the process of carcinogenesis such as cell proliferation and apoptosis, epithelial mesenchymal transition, invasion and metastasis, angiogenesis, immune evasion, cellular crosstalk, cancer cachexia through a variety of biological mechanisms [6, 7]. They can serve as microRNA sponges, interact with RNA binding proteins, regulate gene transcription, modify N6-Methyladenosine, and so on, which are associated with tumorigenesis, development, and patients’ clinical prognosis [6, 7].

Previous studies have confirmed that exo-circRNAs has higher stability and abundance. Their important roles in the occurrence and development of digestive malignancies have attracted considerable attention. Hence, we focused on exo-circRNAs and summarized the update of digestive malignancies associated exo-circRNAs in biogenesis, biological functions, molecular mechanisms, clinical implications, potential applications, and experimental techniques to effectively promote transformation and application in the future. All the exo-circRNAs mentioned in this manuscript are summarized in Table 1.

**Table 1.** Relationship between exo-circRNAs and digestive malignancies.

Cancer type	Exo-circRNA	Level	miRNA sponged	Target/pathways	Functional Phenotypes	Clinical significance	References
Esophageal squamous cell carcinoma	Exo-circ-0048117	Up	miR-140	TLR4	Promote tumor metastasis and polarize macrophages	Remodel microenvironment and modulate progression	[49]
	Exo-circ-0043603	Up				Prognostic biomarker	[140]
	Exo-circ-0026611	Up			Correlate with lymph node metastasis	Prognostic biomarker	[149]
	Exo-circ-0000337	Up	miR-337-3p	JAK2	Decrease in CDDP-resistant	Therapeutic target	[154]
	Exo- circFNDC3B	Up	miR-490-5p	miR-490-5p/TXNRD1	promotes the progression of esophageal squamous cell carcinoma	Play a tumor promotor role	[126]
Gastric cancer	Exo-circNRIP1	Up	miR-149-5p	AKT1/mTOR	Promote proliferation, EMT, migration, invasion	Play a tumor promotor role	[52]
	Exo-circRanGAP1	Up	miR-877-3p	VEGFA	Enhance the migration and invasion ability	Prognostic biomarker and therapeutic target	[53]
	Exo-circ_0004303	Up	miR-148a-3P	ALCAM	Promote the migration and homing	Illustrate the mechanism	[95]
	Exo-circITCH	Up	miR-199a-5p	Klotho	Inhibit the proliferation, migration, invasion and EMT	Diagnostic biomarker and therapy targets	[90]
	Exo-circPVT1E	Up	miR-30a-5p	YAP1	Inhibit apoptosis and promote invasion or autophagy	Prognostic predictor for patients receiving DDP therapy	[83]
	Exo-circNEK9	Up	miR-409-3p	MAP7	Promote proliferation, migration, invasion and motility	Promote the migration and invasion	[104]
	Exo-circSHKBP1	Up	miR-582-3p	HUR/VEGF	Promote cell growth and suppress HSP90 degradation	Diagnosis, prognosis biomarker and therapy targets	[122]
	Exo-circ_0065149	Up			a superior value of sensitivity and specificity	Diagnostic biomarker	[138]
	Exo-circ-KIAA1244	Down			correlate with TNM stage and lymphatic metastasis	Diagnosis, prognosis biomarker	[139]
	Exo-circ-0000419	Down			associate with Borrmann type and differentiation grade	Diagnosis, prognosis biomarker	[145]
	Exo-circ-0000260	Up	miR-129-5p	MMP11	Promote cell proliferation, migration, invasion, adhesion and inhibit apoptosis	Regulate CDDP chemoresistance	[153]
	Exo-circ-0032821	Up	miR-515-5p	SOX9	Boost OXA resistance, proliferation, migration, and invasion	Therapeutic target for OXA chemoresistance	[156]
	Exo-circUBE2Q2	Up	miR-370-3p	STAT3	Inhibit autophagy, promote glycolysis and metastasis	Therapeutic target	[111]
	Exo-circ-FBXW7	Down	miR-18b-5p	FBXW7	Increase the oxaliplatin-induced apoptosis and inhibit oxaliplatin-induced EMT	Therapeutic strategy for oxaliplatin-resistant CRC	[160]
	Exo-circ-0039411	Up	miR-136-5p	MMP2	Promote invasion and migration	Prognosis predictor for poor survival	[50]
Hepatocellular carcinoma	Exo-circ-0004277	Up		ZO-1	Enhance the proliferation, migration, and EMT	Novel therapy	[80]
	Exo-circPTGR1	Up	miR449a	MET	Promote migration and invasion	Prognostic biomarker and therapeutic target	[92]
	Exo-circ-0006602	Up			Promote proliferation and invasion	Diagnostic biomarker	[81]
	Exo-circ-005143	Down	miR-331-3p	BAK1	Promote cell apoptosis and arresting the cell cycle	Predictor and therapeutic target	[84]
	Exo-circDB	Up	miR-34a	USP7/Cyclin A2	Promote HCC growth, body fat ratio and reduce DNA damage	Understand the association between adipose tissues and HCC	[78]
	Exo-CircCdr1as	Up	miR-1270	AFP	Accelerate proliferation and migration	Promoting factor and therapeutic target	[94]
	Exo-circ100338	Up		MMP9	Promote proliferation, angiogenesis, permeability, and vasculogenic mimicry formation ability	Therapeutic strategies, diagnostic and prognostic biomarker	[103]
	Exo-circUHRF1	Up	miR-449c-5p	TIM-3	Inhibit IFN-γ, TNF-α secretion and NK cell proportion, infiltration	Contribute to immunosuppression therapy	[116]
	Exo-circ-0004001	Up				Diagnostic biomarker	[141]
	Exo-circ-0004123	Up				Diagnostic biomarker	[141]
	Exo-circ-0075792	Up				Diagnostic biomarker	[141]
	Exo-circ-0070396	Up				Diagnostic biomarker	[142]
	Exo-circ-0028861	Down			Novel diagnostic tool	Diagnostic biomarker	[143]
	Exo-circ-133	Up	miR-133a	GEF-H1/RhoA axis	Promote cancer metastasis	Therapeutic target	[162]
	Exo-circ-0072088	Up	miR-375	MMP-16	Suppress Invasion and Migration	Biomarker for diagnosis and prognosis, therapeutic target	[108]
	Exo- circCCAR1	Up		circCCAR1/PD1 deubiquitination	CD8 T cell dysfunction, resist ance to anti-PD1 immunotherapy	Contribute to immunosuppression therapy	[115]
	Exo-circPDE8A	Up	miR-338	MACC1/MET	Promote the invasive growth	Biomarker for diagnosis and prognosis	[93]
Pancreatic cancer	Exo-circ-IRAS	Up	miR-122	ZO-1	Enhance endothelial monolayer permeability and tumor invasion, metastasis	Indicator for early diagnosis and prognostic prediction	[102]
	Exo-circPDK1	Up	miR-628-3p	BPTF/BIN1	Promote glycolysis	Diagnosis and prognosis biomarker, therapeutic target	[148]
	Exo-circ-0006357	Up				Diagnostic biomarker	[135]
	Exo-circ-0002111	Up				Diagnostic biomarker	[135]
	Exo-circ-0001678	Up				Diagnostic biomarker	[135]
	Exo-circLONP2	Up	miR-17	DGCR8	Enhance invasion and metastasis through modulating the maturation	Prognostic predictor and anti-metastasis therapeutic target	[54]
Colorectal cancer	Exo-circ_0005963	Up	miR-122	PKM2	Promoting glycolysis and drug resistance	Therapeutic target for oxaliplatin-resistant	[55]
	Exo-circ-ABCC1	Up		Wnt/β-catenin	mediate cell stemness and metastasis	Therapeutic target	[56]
	Exo-circIFT80	Up	miR-1236-3p	HOXB7	Promote proliferation, EMT and induce invasion and migration	Therapeutic strategy	[57]
	Exo-circFMN2	Up	miR1182	hTERT	Promote tumor proliferation	Prognostic biomarker and therapeutic target	[58]
	Exo-circ-HMGCS1	Up	miR-34a-5p	SGPP1	Promote proliferation, invasion and suppress apoptosis	Select more reasonable anesthetics	[85]
	Exo-circPACRGL	Up	miR-142-3p	TGF-β1	Promote proliferation migration and invasion, differentiation	Play an oncogenic role in CRC proliferation and metastasis, therapeutic target	[77]
	Exo-circ-0004771	Up			Remarkable Diagnostic Value	Diagnostic biomarker	[136]
	Exo-circ-PNN	Up			Remarkable Diagnostic Value	Diagnostic biomarker	[137]
	Exo-circ-0000338	Up			Intercellular communication	Biomarker for FOLFOX-resistant prediction and therapeutic target	[161]
	Exo-circ-0067835	Up	miR-296-5p	IGF1R	Promote cell proliferation and inhibit cell apoptosis, radiosensitivity	Therapeutic target for radioresistance	[151]
	Exo-circ-RNF121	Up	miR-1224-5p	FOXM1	Regulate tumor progression, promote glucose uptake, cell glycolysis	Therapeutic target	[97]
	Exo-circ-COG2	Up	miR-1305	TGF-β2/SMAD3	Promote proliferation, migration, and invasion	Prognostic and therapeutic target	[147]
	Exo-circTUBGCP4	Up		AKT	promotes vascular endothelial cell tipping and colorectal cancer metastasis	Play a tumor promotor role	[123]
	Exo-circ-0000199	Up	miR-145-5p, miR-29b-3p		Facilitate cell proliferation and inhibit apoptosis	Prognostic biomarker and therapeutic target	[51]
Oral squamous cell carcinoma	Exo-circUHRF1	Up	miR-526b-5p	c-Myc//TGF-β1/ESRP1	Promote tumor proliferation, migration, invasion and EMT	Therapeutic target	[91]
	Exo-circ-0000284	Up	miR-637	LY6E	Enhance the migration, invasion and proliferation abilities	promoting factor for cholangiocarcinoma progression	[79]
Cholangiocarcinoma	Exo-circ-CCAC1		miR-514a-5p	YY1	increased cell leakiness, induces angiogenesis, disrupts vascular endothelial barriers	Diagnostic, prognostic biomarker and therapeutic target	[127]

## 2. Biogenesis and characteristics

### 2.1. The biogenesis and characteristics of exosomes

**Figure 1.**
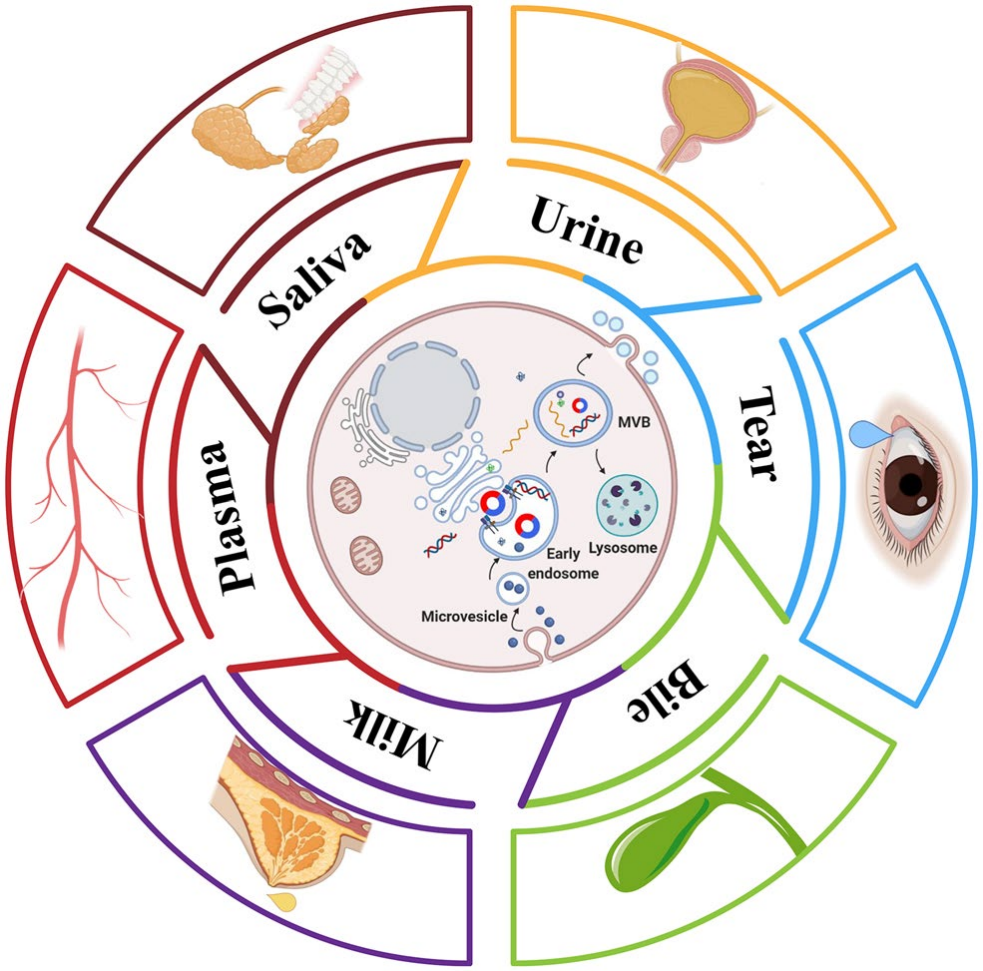
**The biogenesis and secretion of exosome**. The plasma membrane is entrapped into cell to form the microvesicle and mature into early exosome loaded into various kinds of cargoes such as exo-circRNAs and further transfer to multivesicular bodies (MVBs) with Golgi complex. Finally, majority of exosomes are secreted into several body fluids via exocytosis and the other are degraded by lysosome.

Exosomes were originally isolated in the form of vesicles from sheep reticulocytes by Johnstone in 1987, which carried the characteristics of reticulocyte membrane, thought only to be a kind of ‘waste’ from the maturation of erythrocyte [8]. Further, it has been confirmed that exosomes are a kind of spherical, flat or cup-shaped vesicles with a diameter of about 30–150 nm and a double-layer phospholipid membrane structure [9–11]. As shown in Figure 1, exosomes originating from the process of internalization, entrap the plasma membrane to form the early endosome, which gradually mature into late multivesicular bodies (MVBs) by interacting with the Golgi complex [12]. MVBs grasp and incorporate recycled proteins not only from coated pits, but also receive proteins directly from the Golgi complex. With this process, other cargos such as miRNA, mRNA, circRNA and DNA are also incorporated into intraluminal vesicles, and then secreted into extracellular spaces by exocytosis [13, 14]. The last mature intraluminal vesicles called exosomes. This progress requires a dynamic interplay *via* Rab and SNARE protein family [15, 16]. Exosomes play a variety of biological functions in body fluids, and are finally discerned and cleared by the mononuclear phagocytic system in the liver, spleen, and kidneys [17].

Exosomes exist and can be detected in different human body fluids, such as serum, gastric juice, urine, saliva, cerebrospinal fluid, bile, milk and tears [18,19] (Figure 1). The special double-layer phospholipid membrane structure effectively makes exosomes avoid degradation from lysosomes and becomes more stable [20]. Meanwhile, exosomes selectively load specific RNA into themselves and ulteriorly deliver their cargoes to adjacent or distant cells to realize multiple biological functions that closely related to various pathophysiological processes [21].

### 2.2. The biogenesis and characteristics of exosomal circRNA

CircRNAs are a class of novel endogenous RNA molecules through ‘back-splicing’ and covalently linked at the 5′ and 3′ ends [4]. As one of the most important information materials, some circRNAs are stable and abundant in exosomes called exosomal circRNAs (exo-circRNAs) [22]. In eukaryotic cells, the biogenesis of exo-circRNAs can be divided into several main ways based on their different genomic origins: exonic circular RNA (generated by intron-pairing-driven circularization and lariat-driven circularization), circular intronic RNA (driven by several short sequences located in specific introns, ciRNA), exon-intron-derived circRNA (exons circularized and intron sequences retained, elciRNA), RBP-driven circularization-derived circRNA, turns precursor derived circRNA [23].

Up to now, the mechanism of exo-circRNAs entering exosomes is still not very clear. However, it has been confirmed that exo-circRNAs can be actively and selectively incorporated into exosomes in the sorting process by endosoma-sorting complex required for transport II (ESCRT-II) [24]. Besides, recent investigations indicate that intracellular miRNAs help exo-circRNAs enter exosomes as well [25]. For example, CDR1-AS, binding to miR-7 and strongly suppressing miR-7 activity, is remarkably downregulated in exosomes, but slightly elevated in cells when miR-7 is ectopically expressed [26].

Contrasted with parental cells, exo-circRNAs are more enriched than linear RNA in exosomes [27]. There are some possible mechanisms accounting for this phenomenon. First, exo-circRNAs are more stable than linear RNAs in exosomes. The particular covalently closed-loop structure of exo-circRNAs escapes degradation by endonuclease, thereby incorporated more into exosomes [28]. The double-layer phospholipid membrane of exosomes acts as a protective umbrella, preventing exo-circRNAs degradation from RNA enzymes and making exo-circRNAs more stable [29]. Some specific sequence features and protein partners may help exo-circRNAs maintain structural integrity as well [25]. Hence, despite the low speed of exo-circRNA synthesis, they can still accumulate in cells with slow division rates [30]. Secondly, exo-circRNAs have a wide range of sources. The expression profile of exo-circRNAs in exosomes is different from their parent cells, indicating that circRNAs positively enter exosomes from other sources [26]. In addition, exo-circRNAs can be eliminated from parent cells by secreting to body fluid *via* extracellular vesicles, which will enrich other cells and platelets and finally create a positive cycle to input exo-circRNAs continuously [28, 31]. For these reasons, exo-circRNAs are more enriched and stable than linear RNA in exosomes, which allows crucial biological functions and molecular mechanisms.

### 2.3. Isolation and purification

#### 2.3.1. Exosomes isolation

Several methods have been equipped for extracting and purifying exosomes and circRNAs from various mediums. The most common way to extract exosomes is ultra-centrifugation for a variable period (2–5 h) at 100,000 g, which is suitable for proteomics researches for higher protein purity. However, the restrictions of starting length and the viscosity of the initial samples may cause side effects to the outputs and this way always needs much larger sample size. Recent years, sucrose density gradient ultracentrifugation is becoming more and more commercialization with advance fittings to increase the precipitation and the specificity of products.

In line with this, several novel size-based techniques are using for exosome isolation and ultrafiltration is the most common technology. Ultrafiltration can retain the exosomes according to the size and molecular weight cut off (MWCO) of the filtration membrane but fine particulate matter may clog the filter unit and loss some exosomes inevitably [32]. Some commercially available kits such as ExoMir Kit from Bioo Scientific, ExoQuick and total exosomes isolation reagent (TEI) from System Biosciences company and Invitrogen company are convenient and time-saving to isolate exosomes through precipitation solutions [33, 34]. Moreover, size exclusion chromatography (SEC) not only can boost the yield but also maintain its biological functions, which may make exosomes isolation become more standardized and realistic [10].

Immunoaffinity capture-based techniques rely on the special antibody to bind the antigen on the surface of the exosome and attach exosomes to the plate, magnetic beads, resins, and microfluidic devices, which allow for isolating exosomes from a specific source [35]. For example, Abs recognizing tumor associated antigens can be coated on beads and used for capturing the tumor associated exosomes [36]. Although these methods can get higher purity of exosomes, the yield often is lower than others. Hence, researches always firstly use ultracentrifugation or ultrafiltration to enrich exosomes in those complex fluids and further choose this way [37]. More interestingly, Chen et al. proposed a new method using negative pressure oscillation and double coupled harmonic oscillator-enabled membrane vibration to purify exosomes more efficiently, called ultrafast-isolation system (EXODUS) [38]. Their two coupled oscillators generate dual-frequency transverse waves on the membranes, further enables to outperform other isolation techniques in speed, purity and yield.

More methods for immunocapture are developing to elevate the accuracy for separating the exosomes. The above methods can be synergistic and complementary when used in combination to increase the efficiency of exosome extraction.

#### 2.3.2. Purification and quantitation of exo-circRNAs

The methods of RNA extraction are quite mature. TRIzol LS reagents (Ambion, Carlsbad, CA, USA) is a frequently-used and effect theory to extracted exosome RNA. Emerging researches have proposed some innovations based on TRIzol LS reagents. For example, Hazman et al. used a novel combination of CTAB and Trizol protocols to yield RNA with fully preserved integrity [39]. More interestingly, Beltrame et al. optimized the progress of using RNeasy MiniKits(Qiagen) to get high-quality RNA products from planktonic and sessile cells [40]. RNeasy FFPE kit (QIAGEN GmbH, Hilden, Germany) is more suitable for isolating circRNAs from formalin-fixed paraffin-embedded (FFPE) specimens [41]. Such methods are making exo-circRNA extraction much more easily and intactly.

Real-Time Polymerase Chain Reaction (RT-PCR) is an established and common way to quantitate the exosomal circRNA. It can monitor the accumulation of amplification products through the accumulation of fluorescent signals by adding a fluorescent probe into the reaction system that can indicate the reaction process. The results are determined by fluorescence curves, and can be quantified by cycle threshold (Ct) values and standard curves, which have lots of advantages such as convenient, economical, precise and just needs a little exo-circRNA. However, it is easy to be disturbed by residual protein as well as chemical contaminants and difficult to detect those low abundant exo-circRNAs with small expression differences of 2-fold or lower [42].

Droplet Digital PCR (ddPCR) is the third generation of PCR appeared in the recent years, which can determine the absolute number of target molecules in a single copy. ddPCR offers a nano-liter-scale PCR because it divides the reaction into 20,000 droplets in Bio-rad *via* microfluidics and water-in-oil droplets in order to dilute the background DNA and proteins, which not only improves the precision and sensitivity compared to RT-qPCR, but also permits researches to quantitate exo-circRNAs directly using Poisson statistics rather than calibration curves [43]. Nevertheless, the number of droplets and high accuracy system require enough exo-circRNAs and high specific primers. With the improvement of purification accuracy, ddPCR will play dramatic roles in exo-circRNAs’ quantitation.

## 3. Biological functions and molecular mechanisms

### 3.1. The molecular mechanisms of exo-circRNAs

**Figure 2.**
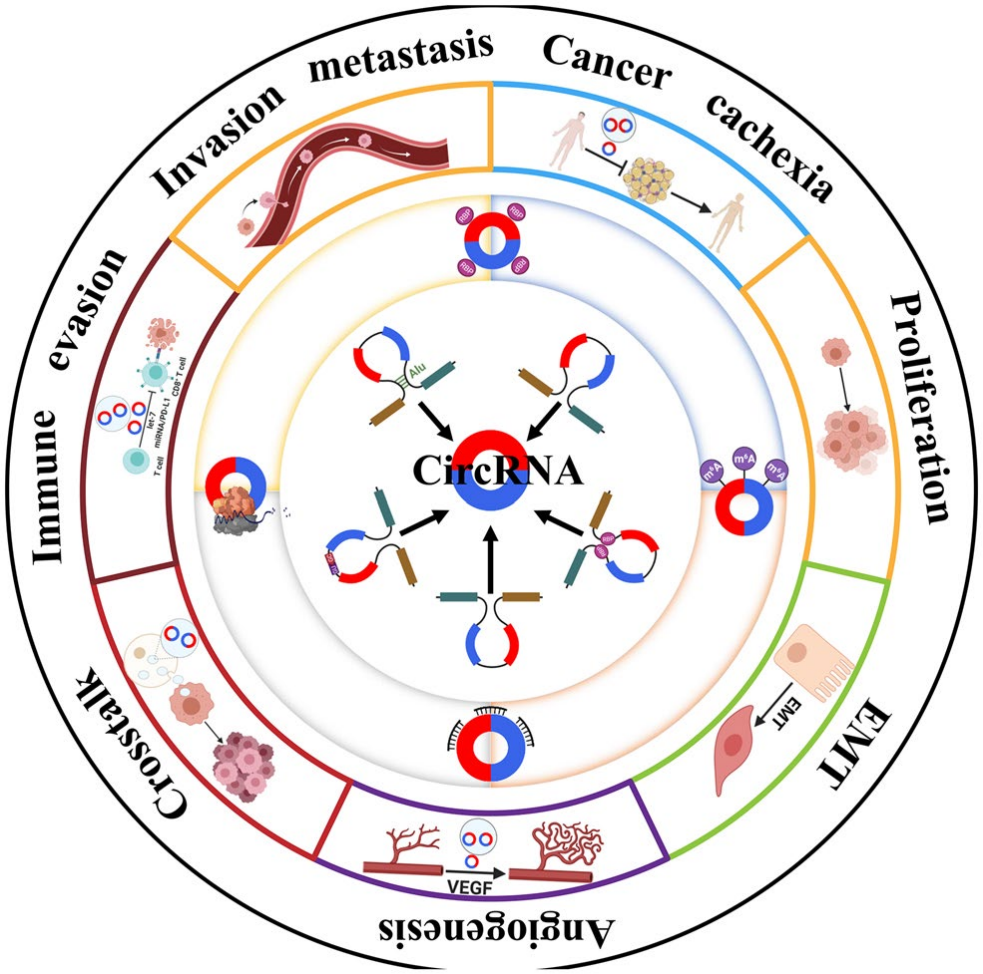
**Biogenesis, molecular mechanisms and biological functions of exo-circRNA**. Based on their different genomic origins, exo-circRNA can be divided into exonic circular RNA; circular intronic RNA; exon-intron-derived circRNA; RBP-driven circularization-derived circRNA; turns precursor derived circRNA. They can affect a variety of cell biological functions via acting as miRNA sponges, binding to RNA binding proteins (RBPs), regulating protein transcription and N6-Methyladenosine Modification.

Exo-circRNAs are involved in various physiological and pathological processes by mediating communication between different cells, especially in the occurrence and progression of cancer. Then, how do exo-circRNAs work? Nowadays, more and more exo-circRNAs have been found to regulate gene expression at transcriptional, posttranscriptional and translational levels. With the increasing recognition of exo-circRNA, the molecular mechanisms of exo-circRNAs are gradually unveiled, such as acting as miRNA sponges, binding to RNA binding proteins (RBPs), regulating protein transcription and N6-Methyladenosine Modification (Figure 2) [44].

#### 3.1.1. Acting as miRNA sponges

The structural basis for exo-circRNAs as miRNA sponges in regulating downstream miRNAs involves the existence of seed sequences. Exo-circRNAs isolate miRNAs from their target mRNAs through their conjunct incomplete complementary base pairing sequences (seed sequences) and then indirectly suppress the activity of miRNAs and regulate downstream target expression, which is called ‘miRNA sponge effects’ [45, 46]. This mechanism that exo-circRNAs and downstream target compete with each other for miRNAs is also known as competing endogenous RNAs (ceRNA).

Exo-circRNAs/miRNAs axis correlate with various regulatory pathways. When clearly identified, it can significantly help to comprehend the instruction of signal transduction. For instance, exo-CDR1as, containing more than 70 binding sites of miR-7, functions as a miR-7 sponge and directly regulates cell growth and invasion in gastric and colorectal cancer [29, 47]. Exo-circ50547 serves as a sponge for miR-217, leading to a decrease in its expression level, and to promote the progression of GC cells [48]. In esophageal squamous cell carcinoma (ESCC), hypoxia can stimulate esophageal squamous cells to secrete hsa-circ-0048117-rich exosomes. Exo-circ-0048117 can absorb miR-140 and polarize macrophages secreting Arg1, IL-10 and TGF-β, furtherly promoting the ability of tumor metastasis [49]. Likewise, upregulated exo-circ_ 0039411 derived from hepatocellular carcinoma (HCC) enhances cell invasion and migration *via* sponging miR-136-5p and facilitating MMP2 expression [50]. Plasma exo-circ_0000199 facilitated cell proliferation and inhibited apoptosis in oral squamous cell carcinoma (OSCC) by interacting with miR-145-5p and miR-29b-3p [51]. Similarly, exo-circNRIP1, exo-circRanGAP1, exo-circLONP2, exo-hsa_circ_0005963, exo-circ-ABCC1, exo-circIFT80, exo-circFMN2, exo-circFNDC3B, exo-circSTRBP have been shown to regulate cell biological function in digestive malignancies by acting as miRNA sponges [52–60].

#### 3.1.2. Binding to RBPs

RBPs are a broad kind of proteins with the specificity of interacting with RNA molecules. Exo-circRNAs and RBPs interact with each other in the progress of their formation. More than one hundred thousand exo-circRNAs potentially associate with the RBPs EIF4A3 *via* TDP43 at the flanking sequences of exo-circRNAs [61]. Increasing researches have demonstrated that RBPs correlate with exo-circRNAs junctions and play crucial roles in their splicing, processing, folding, stabilization, and localization [62]. The activity of RBPs and exo-circRNAs act as scaffolds for protein complexes and downstream messengers connecting the intracellular and extracellular environment.

Exo-circRNAs and RBPs interact with each other in the progress of their formation. Quaking (QKI) is a class of RBPs belonging to STAR family, containing three major molecules called QKI-5, QKI-6, and QKI-7. Simon et al. has convinced that QKI binding elements in introns are essential for the formulation of circRNAs using a novel color reporter construct [61]. In addition, QKI can make the circle-forming exons closer to each other in order to produce new circRNAs as well.

Moreover, RBPs are a necessary part for exosomes to transform exo-circRNAs [56, 62]. Latest evidences confirmed that RBPs are a necessary part for compressing relatively huge nucleic acid structures into the interior spaces of exosomes, which may involve in the exo-circRNAs transmission [62, 63]. RBPs act as the intracellular inducers for exo-circRNAs accurately loading into exosomes at target cells and vital factors for promoting exo-circRNAs transmission in their parent cells as well [64, 65]. Exo-circ_0040573, exo-circ_0040571 regulate the expression of the tumor suppressor p53 in target cell following the transmission of exosomes [63]. Not only that, p53 can conversely affect the synthesis of these exo-circRNAs in parent cells *via* mediating the activity of QKI, which reveals that the expression of RBPs regulated by parent cell can influence the biogenesis of exo-circRNA by excretion [61].

Furthermore, exo-circRNAs interact with miRNAs by RBPs assistance to involve in post-transcriptional regulation in several approaches. Argonaute protein 2 (Ago2) is a typical RBP that functions as a central factor for circRNAs to combine miRNA to regulate the mRNA generation [66]. For example, the degradation of exo-circCdr1as was dependent on Ago2 mediated miR-671, independently of heterochromatin formation [67].

#### 3.1.3. Regulating transcription and protein translation

Transcription is an essential biological process of gene expression including initiation, elongation and termination. Recent studies displayed that circRNAs regulate the process of transcription at the stages of initiation and elongation. U1 snRNA is a core component to promote the formation of the first phosphodiester bond by Polymerase II in the initiation step of transcription [66]. There is enough evidence to say that various exon-intron circular RNAs contain the site to bind U1 snRNA in order to influence the transcription. Li et al. have found that 111 circRNAs associated with Polymerase II by U1 snRNA, such as circEIF3J and circPAIP2. CircEIF3J and circPAIP2 can mediate the expression of their parental genes *via* upregulating the transcription factor EIF3J and PAIP2. However, blocking the U1 snRNA binding site not only can reduce the combination of U1 snRNA and two circRNAs but also decrease the interactions between Polymerase II and two circRNAs as well as the promoter sequences, which clearly showed the function of circRNAs in transcription [67].

Likewise, a group of circRNAs take part in the elongation of transcription. The phosphorylation of Polymerase II is a crucial step for elongation progress. Intronic circular RNAs ankrd52 (ci-ankrd52), derives from the intron in its parental gene and increases the elongation of its parental gene by interacting the phosphorylated Polymerase II [68]. Similar effects have been convinced in ci-mcm5, and ci-sirt7 as well [64]. However, relative research about the correlation of exo-circRNA and transcription is still few. ElciRNA and ciRNA are two important sources of exo-circRNA and the association of transcription and exo-circRNAs needs to be explored in the future.

Translation from mRNAs is mediated by the key structures of initiation codon (AUG), sequence and open reading frames (ORFs), poly(A) tails and 5′ end cap of mRNAs. Although lack of poly(A) tails and 5′ end cap and the majority of circRNAs are considered as non-coding RNAs, some exo-circRNAs containing with opening reading frames can function as the mRNAs to direct the synthesis of downstream proteins under some circumstances [65]. Some factors including internal ribosome entry sequences (IRES), N6-methyladenosine (m6A) modifications and specific sequences in the untranslated region (UTR) of circRNAs have been revealed to induce circRNA translation [69]. For example, exosomal circALTOs can function as a translation template and generate ALTO proteins *via* N6-methyladenosine (m6A) modifications in cells. The transcript levels will be downregulated by specifically co-transfecting circALTO1/2 plasmids and BSJ-specific siRNAs or ALTO-targeting siRNAs [70]. Liang W et al. suggested that circ-0004194 was overexpression in HCC tissue, which had a particular IRES and encoded a 370 amino acids active protein termed as β-catenin-370aa. CircPPP1R12A contained a special ORF and encoded a functional protein called circPPP1R12A-73aa, which promoted the metastasis of colon cancer [71]. Regrettably, the roles of circ-0004194 and circPPP1R12A in exosome are unknown yet.

Recent study have demonstrated that unmodified exo-circRNAs can diminish or even avoid the immune response and stably translate and synthesize proteins in mice by bypassing the RNA sensors such as RIG-I and toll-like receptor (TLR), which depends on the high purity of exo-circRNAs [72]. More exciting thing is that novel engineering approaches are performed to generate exogenous circRNAs for potent and durable protein expression [73]. Although the research of circRNAs’ transcription and translation functions are a hot spot at present, exo-circRNAs in transcription and protein translation and their molecular mechanism are still not very unclear. Further researches need to explore more mechanism and function in the side of exo-circRNAs transcription and translation and provide more theoretical supports for digestive malignancies treatment.

#### 3.1.4. N6-Methyladenosine modification

N6-methyladenosine (m6A) modification refers to the methylation modification that exists on the sixth nitrogen atom of adenine, which is the most prevalent internal modification of circRNA. Compared to mRNA, m6A modification has the same regulators with mRNA, including m6A Writers, Readers and Erasers, while the function of m6A modification in exosomal circRNA is still indistinct [74].

Based on existing research, the maladjusted level of m6A modification in exo-circRNA can affect multiple stages of metabolism progression. For example, it is worth noting that exo-circ_00085429 revealed having a high level of m6A methylation. METTL3 and ALKBH5 are two RNA methyltransferases, which regulated the m6A methylation level of exo-circ_00085429. Inhibiting METTL3 or overexpressing ALKBH5 can significantly facilitate the downstream miR-185-5p/RANK axis and reverse osteoclast differentiation and bone resorption induced by exo-circ_0008542, suggesting m6A methylation plays a critical role in posttranscriptional regulation [75].

Furthermore, the aberrant level of exo-circRNA also influences the m6A modification of downstream molecules *via* m6A modification regulators. It has been convinced that exosomal circ_0072083 derived from resistant cells promotes temozolomide resistance in sensitive glioma cells *via* miR-1252-5p/NANOG signal pathway. ALKBH5 is a representative m6A demethylase acting on mRNA. Exosomal circ_0072083 can upregulate ALKBH5 expression, mediating demethylation of NANOG mRNA 3’UTR, contributing to promote NANOG mRNA stability [76].

Recent studies proved that M6A modification suffice to blunt the immunogenicity response and extend circRNAs’ half-life period, making circRNA more stable. However, as a common modification in exo-circRNA, the connection between N6-Methyladenosine modification and exo-circRNAs is still mysterious, which has broad prospects for future development and application.

The delivery of exo-circRNAs between normal and tumor cells have aroused intense interest in field of tumor mechanisms. Despite great progress, there are still many challenges in the research on the mechanisms of exo-circRNAs, and their practical clinical application.

### 3.2. The biological functions of exo-circRNAs in digestive malignancies

Exo-circRNAs are the significant players between exosomes and tumor cells. A growing number of evidences have proven the representative function of exo-circRNAs in the physiological state of maternal cells, which further mediates cellular responses after captured by recipient cells. Recent reports have shown that exo-circRNAs are tightly related to tumor oncogenesis as well as development and summarized as follows (Figure 2).

#### 3.2.1. Cell proliferation or apoptosis

Extracellular vesicles have been annotated to reshape host environment to generate a favorable medium for tumor growth and proliferation. Accumulating studies point out that exosomes and exo-circRNAs are the key parts of cell proliferation and apoptosis. For example, it has been shown that circFMN2, derived from plasma exosomes, highly expressed in colorectal cancer (CRC) cell and promoted cell proliferation by decreasing the percent of cells in G_0_/G_1_ phase as well as increasing in G_2_/M phase. However, knockdown exo-circFMN2 remarkably inhibited cell proliferation *in vitro* by functional experiments, which suggested its crucial role on tumorigenesis [58]. Likewise, exo-circRNAs also can regulate cell proliferation by acting as sponges for miRNAs. Exo-circPACRGL is remarkably upregulated and playing an oncogenic role in in CRC cell, which promotes CRC cell proliferation by mediating miR-142-3p/miR-506-3p-TGF-β1 axis. Conversely, there is an opposite result occurred in the circPACRGL-knockdown CRC cells, suggesting the vital role of exo-circPACRGL in cell proliferation [77]. Notably, circRNA produced by normal hepatocytes also can influence the process of cancer. CircDB is derived from adipose tissues *via* exosomes, which is elevated in HCC cell and plasma. The upregulated exo-circDB acts as a sponge of miR-34a and manipulates the USP7/Cyclin A2 signaling pathway. Overexpression USP7 significantly decreases the ubiquitination of various proteins such as cyclin A2 and facilitates cell cycle transition from G_2_ to M phase, which finally promotes HCC cell growth [78]. Others like exo-circ0000284 in cholangiocarcinoma, exo-circ-0004277 and exo_circ_0006602 in HCC, elevate the cell proliferation *via* similar mechanisms, affecting the progress of several digestive malignant tumors [79–81].

Moreover, exo-circRNAs also have exceptional ­capacity to regulate cell apoptosis. Upregulated exo-circ_0004658 derived from macrophage-derived exosomes overexpressing RBPJ can suppress HCC cell proliferation and promote apoptosis *via* competitively binding miR-499b-5p and increasing the expression level of JAM3. However, apoptosis in HCC cells was inhibited when exo-circ_0004658 was downregulated, which may help to select more reasonable anesthetics for HCC patients in the future [82]. Furthermore, circIFT80 was overexpression in CRC serum exosomes, playing a vital role in expediting the growth of CRC cell by arresting the cell cycle and inhibiting cell apoptosis through miR-1236-3p/HOXB7 signal pathway *in vivo* and *vitro* [57]. In GC, exo-circPVT1E is overexpression in the plasma exosomes but downregulated in DDP-sensitive GC serums, which modulates the progress of apoptosis *via* by suppressing miRNA-30a-5p in GC cells [83]. Furthermore, exo-circ-005143 [84] in HCC, circ-HMGCS1 [85] in CRC, exo-circ_0001190 [86] in GC, enable to play an important role in regulating apoptosis by analogical mechanism as well.

#### 3.2.2. Epithelial mesenchymal transition

Epithelial mesenchymal transformation (EMT) is a cellular dynamic process of de-differentiation in which epithelial cells transform into mesenchymal cells, furtherly losing their talent behaviors and getting some oncological properties involving cancer motility, invasion, metastasis, therapeutic resistance and recurrence [87, 88]. Hundreds of exo-circRNAs have been identified that regulated the process of EMT in cell growth and differentiation, both purposefully synthesized and regulated by cell type specific mechanisms [89]. In GC, the level of circ-ITCH was lower in GC tissues and serum-derived exosomes, acting as a tumor suppressor gene and influencing EMT progress. However, knockdown of exo-circ-ITCH can increase the expression of EMT-related mesenchymal markers like N-cad, Vimentin, MMP9, conversely inhibiting cell growth, invasion and migration, which implies the important role of exo-circ-ITCH in EMT [90]. Likewise, exo-circNRIP1 functioning as a promoter of EMT, alters cell metabolism and promotes GC development as well as invasion *via* acting as a sponge for miR-149-5p through AKT1/mTOR axis. Whereas, if co-transfecting exo-circNRIP1 with a miR-149-5p inhibitor into GC cells, the mesenchymal cell markers can be downregulated, suggesting that exo-circNRIP1 is a key factor of EMT [52]. Moreover, in the oral squamous cell carcinoma, Wang et al. demonstrated that elevated exo-circUHRF1 stimulated the progress of EMT by decreasing the expression level of epithelial marker E-cadherin and enhancing mesenchymal markers N-cadherin, Vimentin, which promoted tumor growth and correlated with the poor prognosis in patients [91]. In addition, exo-circ-0004277, exo-circPTGR1 in HCC regulate the progress of EMT and act unique roles in these tumors as well [80, 92].

#### 3.2.3. Invasion and metastasis

Exo-circRNAs can regulate tumor invasion and metastasis *via* the mechanisms of miRNA sponges and EMT. The cancer cells with highly invasive characteristics always can efficiently regulate various signal pathways of cellular metabolism. Elevated exo-circPDE8A derived from pancreatic ductal adenocarcinoma (PDAC), promotes lymphatic invasion *via* stimulating MET by miR-338/MACC1/MET pathway [93]. CircCdr1as derived from HCC cells’ exosomes, was significantly upregulated in cell lines and tissues, accelerating proliferative and invasive abilities and regulating the biological functions in the pathological progressions by sponging miR-1270 and elevating AFP [94]. In CRC, exo-circPACRGL upregulated in CRC cells and promoted CRC invasion, as well as differentiation of N1 to N2 neutrophils *via* miR-142-3p/miR-506-3p-TGF-β1 axis, which dramatically influenced and contributed to human CRC treatment [77]. In GC, Wang et al. confirmed that exo-circITCH was downregulated in GC cell, tissue, exosomes, which was tightly related to invasion depth and enhanced cell invasion *via* promoting the progress of EMT, indeed [90]. Exo-circ_0004303 derived from gastric cancer, interacted to the ability of migration and invasion of human adipose-derived mesenchymal stem cells by acting as a sponge for miR-148a-3p [95]. Furthermore, exo-circ_0020256 in cholangiocarcinoma [96], exo-circ-RNF121 in CRC [97], exo-circ-SFMBT2 in esophageal cancer [98], exo-circ_0061395 [99], exo-circ_0046600 [100] in HCC, exo-circ_0000069 in PDAC [101], mediate cell invasion by similar mechanisms as well.

Analogously, exo-circRNAs also participate in the process of tumor metastasis. In pancreatic cancer cells, overexpression exo-circ-IRAS in PDAC showed high invasive ability through absorbing miR-122 by transwell chamber migration assay, furtherly increased endothelial monolayer permeability and activated tumor invasion such as liver metastasis [102]. Similarly, Huang et al. demonstrated that exo-circ100338 was remarkably lower in HCC cell and enhanced the ability of invasiveness, angiogenesis, while knocking down the exo-circ100338 could arrest the cell cycle and reduce the metastatic abilities of HCC cells, which may be an independent risk biomarker for monitoring distal metastasis [103]. The exosomal circ0000284 in cholangiocarcinoma [79], exo-circNRIP1, exo-circNEK9, exo-circNHSL1 in GC [52, 104, 105], exo-circLONP2, exo-circ-ABCC1, exo-circIFT80 in CRC [54, 56, 57], exo-circANTXR1 in HCC [106], also have been reported their metastatic ability.

Interestedly, some exo-circRNAs certainly both influence tumor cell invasion and metastasis. Recent findings showed exo-circ-0030167, secreted from bone marrow mesenchymal stem cells (BM-MSCs), also could disturb the invasion and metastasis functions of pancreatic cancer cells by sponging miR-338-5p as well as increasing the level of Wif1 and further inhibiting the Wnt8/β-catenin signal pathway [107]. Likewise, circ-0072088 derived from HCC exosomes regulated the degradation of miR-375 and elevated MMP-16, furtherly inhibiting the invasion and metastasis ability of HCC, which subsequently was demonstrated in the *vivo* experiment as well [108]. These explorations suggest that exo-circRNAs also contribute to induce the characteristics of tumor invasion and metastasis as well. These examples imply that exo-circRNAs play an active role in tumor invasion and provide a neoteric rationale for the investigation of tumor metastasis.

#### 3.2.4. Cellular crosstalk

In the past decade, much more attentions have been paid on the potential value of circRNAs packaged within exosomes in controlling cell-cell crosstalk. Exosome is a mediator that uptakes and provides a shield for vulnerable circRNAs degradation by RNases. Malignant tumors seem to exploit this system for cell-to-cell communication in carcinogenesis. For example, exo-circ_100284 in HCC derived from arsenite-transformed cells could shuttle from tumor cells *via* exosomes and was confirmed to transmit from arsenite-transformed human hepatic epithelial cells and result in the malignant transformation, which induced the acceleration of the cell cycle and promoted proliferation *via* miR-217. Next, knockdown of exo-circ_100284, reduced expression level of exo-circ_100284 in exosomes derived from transformed cells, furtherly blocked the accelerated cell cycle and reduced proliferation and malignancy detected by flow cytometry [109]. Similarly, exo-circ_00074854 can transferred from HCC tumor cells to macrophages *via* exosomes, which inhibits the progress of macrophage M2 polarization *in vitro*. This effect further suppresses HCC cell migration, invasion and EMT *via* binding HuR(an RNA-binding protein in HCC) [110]. This homologous communication can also be found in exo-circ-0000284 in cholangiocarcinoma [79], exo-Cdr1as [94] in HCC, exo-circUBE2Q2 in GC [111]. All these results show that cellular crosstalk is an indispensable part of tumorigenesis.

In contrast, circ-0051443 was secreted from healthy cells to HCC cells *via* exosomes and acted as a regulator of intercellular communication, the level of which is enriched by approximately four-fold in the exosomes. Exo-circ-005143 may be a tumor suppressor targeted by miR-331-3p and was downregulated in HCC tissue compared to the normal. In the upregulation cell, the cell cycle was arrested in the G_0_/G_1_ phase and the proportion of apoptosis was remarkably elevated, further inhibited the tumor growth *in vivo* [84]. Cancer-associated fibroblast (CAF)-derived exosomes also play a key role in cellular crosstalk. In GC, Shi et al. proved that CAF delivered exo-circ_0088300 to GC cells by exosomes, which sponged miR-1305 and stimulated GC cell proliferation, growth, and invasion [112]. These cases point out that normal cells can influence and regulate tumorigenesis *via* secreting exosomes contained functional exo-circRNAs, which is a momentous part of cell-cell crosstalk.

Therefore, we suppose that exo-circRNAs mediate bidirectional communication between tumor cells and surrounding environment and are central effectors to shape the ever-evolving tumor microenvironment. Since exo-circRNAs have been implied as an authentical way for tumor information transfer between distal malignant and surrounding cells, they will promisingly contribute to the epigenetic and biological properties of cancers.

#### 3.2.5. Immune evasion

The tumor-driven microenvironment and communication system are oriented to the benefits of tumor growth and invasion. To weak even silence the effects of immune response, circRNAs carried by exosomes induce the modulation and dysfunction of different immunocytes and diverse immune responses [113]. For example, in HCC, upregulated exo-circTMEM181 helps to create and maintain an immunosuppressive microenvironment by binding miR-488-3p and elevating the expression of CD39 in macrophages. The overexpression of CD39 accelerates the progress of degrading the ATP to ADP and AMP, further weakening the signal of the immune reaction motivated by eATP in HCC microenvironment [114]. Exo-circCCAR1 acts as a sponge of miR-127-5p to up-regulate its target WTAP, and forms a feedback loop consisting of the circCCAR1/miR-127-5p/WTAP axis. CircCCAR1/miR-127-5p/WTAP feedback loop can promote the growth and metastasis of HCC. Exosomal circCCAR1 can be transferred to CD8^+^ T cells and make PD1 express stably to promote CD8^+^ T cell dysfunction and anti-PD1 resistance in HCC as well [115].

Natural killer (NK) cell is a critical component of innate antitumor immune response. Zhang et al. reported that upregulated exosomal circUHRF1 correlated with lower NK cell proportion and tumor infiltration *via* suppression of miR-449c-5p and promoting the expression of TIM-3, which provided a novel therapeutic target for HCC patients [116].

Macrophages also are important type of innate immune. Exo-circRNAs have been reported to regulate macrophage polarization to aid tumor progression and immune escape [117]. For example, under hypoxic conditions, exo-circ0048117 originating from esophageal squamous cell carcinoma cells exhibits the ability to traverse to macrophages, inducing M2-type polarization [49]. In addition, exosomal circATP8A1 promotes GC progression by inducing the M2 polarization of macrophages through the STAT6 pathway [118]. Also, exo-circPOLQ can enter macrophages and generate CRC metastatic nodule formation by facilitating M2 macrophage polarization [119].

With the features of accommodating immunologic function, exosomes and their exo-circRNAs will serve as dependent noninvasive strategy and more and more exo-circRNAs need to be excavated in the future.

#### 3.2.6. Angiogenesis

Tumor-derived exosomes also trigger cell differentiation to carry pro-angiogenic and pro-invasive characteristics [120]. The broad interests and increasing attention aimed at angiogenesis related exo-circRNAs have also pioneered a new field for assessing the risk of tumor distal metastasis. Vascular endothelial growth factor (VEGF) is a key mediator of angiogenesis in malignant tumors, which is always overexpression by hypoxia and oncogene expression such as circRNAs [121]. In gastric cancer, the level of exo-circSHKBP1 is higher than the normal, regulating the miR-582-3p/HUR/VEGF axis and enhancing the stability of VEGF mRNA, further promoting the peculiarity of angiogenesis and migration *in vivo*, which may be an alternative diagnostic and therapeutic biomarker [122]. Colorectal cancer cells produce exo-circTUBGCP4, which promotes vascular endothelial cell tilting through activation of Akt signaling pathway, further improving angiogenesis and tumor metastasis [123]. On the other side, tight junction protein and adhesion protein contributes to the regulation of barrier property and maintain dynamic tissue integrity, playing important parts in endothelial barrier function and angiogenesis [124, 125]. Exo-circRNAs also directly and indirectly regulate the expression of these proteins to affect the progress of angiogenesis. Exo-circ100338 is overexpressed in HCC cell and downregulated the level of tight junction protein ZO-1 and adhesion protein VE-Cadherin, which not only disrupts the tight junctions and strengths the invasive ability but also stimulates proangiogenic activity and angiogenesis [103].

On the contrary, a variety of exo-circRNAs play critical roles in inhibiting angiogenesis. Exo-circFNDC3B derived from CRC exosomes was downregulated in CRC cell and tissue, which decreased tumor growth, angiogenesis and liver metastasis by regulating miR-97-5p/TIMP3 signal pathway. Upregulated exo-circFNDC3B and miR-97-5p can reversely promote the process of angiogenesis, hinting that exo-circFNDC3B can become a new targeted therapeutic site [126]. Likewise, exo-circCCAC1 in cholangiocarcinoma [127], exo-circIARS in pancreatic cancer [128], exo-circ_0007334 in CRC [129], exo-circ_0044366 in GC [130], affect the formation of extracellular vesicles in various degree as well.

In a word, the cell proliferation and metastasis are tightly dependent on angiogenesis in most malignant tumors, in which the balance is deemed to be oriented toward stimulating angiogenic factors and restraining endogenous inhibitors of angiogenesis [131]. There is no doubt that the exploration of exo-circRNAs will enter into a new epoch of angiogenic targeted therapy.

#### 3.2.7. Cancer cachexia

Energy metabolism is a key part of tumor development associated with carbohydrate, lipid and proteins, which was regulated and orchestrated not only by carcinogenic proteins but exo-circRNAs, aiming at supplying necessary nutrients for tumorigenesis and development [132]. Circ-RNF121 was overexpression in colorectal cancer secreted from exosome, which prominently regulated tumor progression and facilitated glucose uptake as well as cell glycolysis by acting as a sponge for miR-1224-5p and upregulating FOXM1[97]. Exosomes contain abundant nucleic acids and support an efficient interaction with target cells, which is necessary to clarify the connection between exo-circRNAs and cancer cachexia in order to deepen the comprehensions about tumor metabolic network and decrease the mortality of digestive advanced tumors in the future.

## 4. Clinical applications

**Figure 3.**
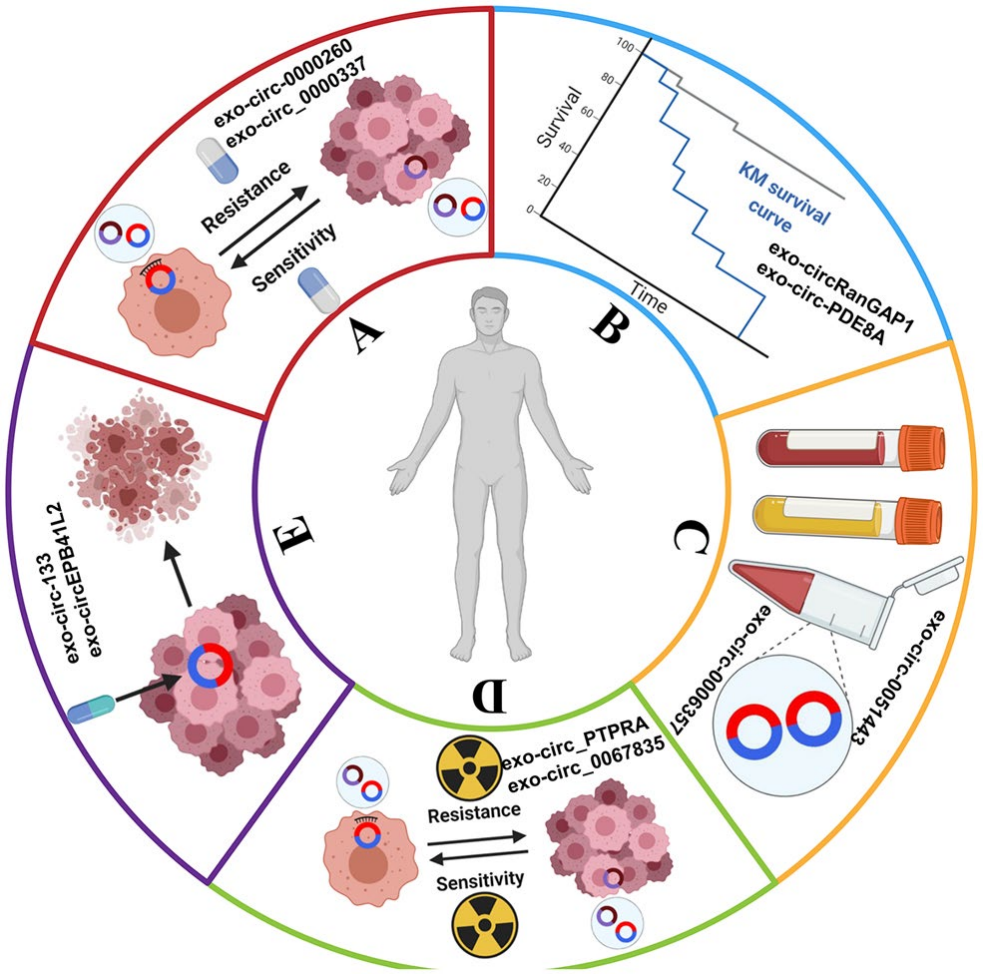
**The clinical applications of exo-circRNAs**. (A) Promote tumor chemoresistance or improve drug sensitivity. (B) Act as prognostic indicators. (C) Act as novel diagnostic biomarkers. (D) Enhance radiation sensitivity. (E) Become treating targets.

Cancer is a multi-stages and multi-factors disease with various selections and mutations. Early screening and diagnosis of cancer is essential, which prominently enhances the odds for establishing individual therapeutic schedule and ameliorates survival quality. We summarized exosomal circular RNAs related to diagnostic and prognostic markers in Supplementary Table 1. As we mentioned before, with the specific bioactive functions, exo-circRNAs contribute to the pathophysiological progress of tumors (Figure 3), which indicates exosomes have the potential to serve as diagnostic biomarkers and therapeutic targets.

### 4.1. Diagnostic molecular markers

Liquid biopsies have the potential to allow physicians to identify tumors with specific mutations in the least invasive way possibly. It has been demonstrated that the level of exosomes in blood of healthy controls has been reported to be 10^10/ml and may increase 3–4 fold in patients with cancer and their exo-circRNAs are stable and detectable, suitable to be novel biomarkers[133]. More importantly, exo-circRNAs are selectively vary in specific tissues and diseases, having the potential to act as a promising tumor biomarker [134]. For example, exosomal circ-0051443 not only suppressed the progress of proliferation, but also showed a distinguished screening capacity for HCC [84]. CircPDAC was encoded from two noncoding RNA located on chromosome 12. A preliminary study has proven circPDAC such as exo-circ-0006357, exo-circ-0002111, exo-circ-0001678 didn’t express in normal pancreatic tissue but upregulated in human PDAC tissues, plasma and exosome, which has been served as a promising biomarker for PDAC [135].

Notably, exosomes and exo-circRNAs especially play important roles in digestive malignancies. For instance, tumor derived exo-circ-0004771 was remarkably upregulated in CRC patients compared to the healthy and downregulated after surgical operation, exhibiting a better AUC of 0.816 (95% Confidence interval, 0.728-0.9) to differentiate stage I/II CRC patients, with a satisfying sensitivity of 81.43% and specificity of 80% in screening CRC [136]. Exo-circ-PNN was upregulated and played a crucial role in the pathogenesis of CRC, having a significant value in screening CRC especially in early stage, whose AUC was up to 0.854 in the training and validation set [137]. Likewise, hsa_circ_0065149 is a satisfied biomarker examined using cell, tissue, plasma and plasma exosomes from 41 healthy volunteers and 39 early gastric cancer patients. Exo-circ_0065149 showed a superior value of sensitivity and specificity were 48.7% and 90.2%, much more than traditional clinical biomarker such as CEA, CA19-9, and CA125, which is a promising biomarker for GC even early gastric cancer in the future [138]. Exosomal circRNAs can be easily detected in serums and it has been reported that exo-circ-KIAA1244, exo-circ50547 in GC [48, 139], exo-circ_0043603 in esophageal squamous cell cancer [140], exo-circ_0004001, exo-circ_0004123, exo-circ_0075792, exo-circ-0070396, exo-circ_0028861 in HCC plasma, exo-circLPAR1 in CRC, have also been verified to be potential reliable cancer biomarker [141–144].

Exosomes exist in almost all physiological fluids and secret into various culture mediums. All these researches illustrate the significant hallmark properties of exo-circRNAs which convince the researchers to explore the power of these molecules in such complex diseases. Furthermore, it will be of great potential to explore more practical exo-circRNAs and application methods in future studies.

### 4.2. Prognostic indicators

Clinicopathologic features are independent prognostic factors and emerging data shows tumor associated exo-circRNAs tightly correlate with clinical outcomes, playing a vital role for regulating tumorigenesis and progression. For instance, upregulated circ-RanGAP1 expression correlated with an advanced TNM stage and worse survival time detected in both GC tissues and exosomes. The GC cell growth and metastasis could be reversed when it was silenced, implying that exo-circRanGAP1 may be a sparkling biomarker [53]. Exo-circ-PDE8A was also upregulated in PDAC plasma, which was found to affect PDAC progression by acting as a sponge for miR-338 and mediating MACC/MET/ERK or AKT pathway. The upregulation of exo-circ-PDE8A significantly correlated with higher risk of lymphatic invasion and higher TNM stage, even lower survival time [93]. Similarly, high expression of exo-circSTRBP in GC is associated with more distant metastasis and advanced TNM stages [60].

Notably, exo-circRNAs have important impacts on the efficacy of patient prognosis in certain tumor associated exosomes also make effects in plasma. Furthermore, exo-circ-0000419 were downregulated in GC patients, tightly associated with clinicopathologic parameters, such as advancer tumor stage, more risk of lymphatic and distal metastasis. The patients with low exo-circ-0000419 had a poor overall survival time (median survival of 39.94 months vs 53.95 months) and disease-free survival (median survival of 35.00 months vs 49.74 months), thereby considered to be an ideal indicator for GC prognosis [145]. In HCC, it has been confirmed that plasma exo-circ_0008043, influenced HCC prognosis through the miR449a/MET pathway. The exo-circ_0008043 elevates the recurrence risk of HCC and decrease the distal survival time, which can be a novel prognostic marker [92]. CircTMEM45A is remarkably overexpression in HCC cell, tissue and plasma exosomes, which associates with several clinicopathological feature and poor prognosis *via* acting as a sponge for miR-665 and elevating IGF2 [146]. Similarly, exosome-secreted circ-0065149, circ-KIAA1244 in GC plasma, exo-circ_0003731, exo-circ_0088030, exo-circ-0070396 in HCC, exo-circCOG2 in CRC [147], exo-circPDK1 in tumor tissues and serum exosomes of pancreatic cancer [148], exo-circ_0026611 in esophageal squamous cell carcinoma are also likely to become independent progSnostic factors [92, 138, 139, 142, 149]. We can monitor the mutation status of malignant tumors based on liquid biopsy in order to predict the risk of recurrence and survival times. In conclusion, exo-circRNAs will reap huge fruits on supervising tumor progress and predict the outcome in order to guide the regimen.

### 4.3. Radiation resistance and sensitivity

At present, radiation therapy especially adjuvant radiation thearpy occupy a key position in the predominant treatment for digestive cancer. Unluckily, insensitivity to radiotherapy leads to poor efficacy and prognosis for patients. Currently, exo-circRNAs have been suggested to enhance radiation-sensitivity in the process of constant observation and exploration. In CRC, exo-circ_PTPRA can improve cell radiosensitivity and inhibit tumor growth *via* acting as a ceRNA to upregulate SMAD4 level by targeting to miR-671-5p, which indicates that exo-circ_PTPRA has the potential to become a novel modifier for clinical adjuvant therapy [150]. Circ_0067835 was remarkably upregulated in CRC tissues and it has been identified that plasma exosomal circ_0067835 was elevated in CRC patients received radiotherapy compared with those untreated. Moreover, upregulated exo-circ_0067835 promoted cell proliferation, cell cycle progression, and suppressed apoptosis and radiosensitivity by sponging miR-296-5p and enhancing IGF1R while this function can be reversed by knocking down exo-circ_0067835 itself, which implies that exo-circ_0067835 may be a new light for improving CRC treatment [151]. Circ_0002130 was more than 8 folds significantly overexpression in exosomes secreted from pancreatic cancer cell after radiation and exo-circ_0002130miR_4482-3p/NBN interaction pathway may be the potential axis regulating cell proliferation [152]. Collectively, we believe that exo-circRNA has the prospect to be a breakthrough to radiation resistance and more methodological development needs to be designed in this subject.

### 4.4. Drug resistance and sensitivity

Chemotherapy is a critical method for malignant tumors while chemo-sensitive cells may gradually transform into chemo-resistant cells during the therapy, leading to a bad therapeutic effect and poor prognosis. Some dysregulated exo-circRNAs provide key indicators to tumor chemotherapy *via* regulating several vital pathways. It has been demonstrated that overexpression exo-circ-0000260 promotes CDDP resistance in GC tissue and plasma by acting as a sponge for miR-129-5p and activating MMP11, which can be reversed *via* inhibiting miR-129-5p or knocking down MMP11 [153]. In line with this, Zang et al. proved that elevated exo-circ_0000337 promoted tumor growth and CDDP resistance *via* downregulating miR-337-3p and improving JAK2 in esophageal cancer *in vivo* and *vitro* [154]. Besides, exo-circ_0006174 [155] in CRC, elevated exo-circ-PVT1[83], exo-circ-0032821 [156], exo-circPRRX1 [157] in GC, exo-circZFR [158] in HCC present similar characteristics in promoting tumor chemoresistance.

Alternatively, several exo-circRNAs play critical roles in improving drug sensitivity. For example, exo-circ-G004213 was downregulated in HCC and had the capacity to lead resistant cells sensitive to cisplatin, which was proven to associate to the distal survival time of HCC patients and provided a new theory of the process of chemotherapy [159]. In CRC, downregulated exo-circ-FBXW7 associated with oxaliplatin-resistant and higher exo-circ-FBXW7 could reverse resistance to oxaliplatin, inhibited oxaliplatin-induced epithelial-mesenchymal transition, and suppressed oxaliplatin efflux *in vivo* and *vitro* by directly binding to miR-128-3p [160]. It has been identified that exo-circ_0000338 played an important role in the development of CRC by targeting miR-217 and miR-485-3p *via* drug sensitivity assay and cell cycle analysis. However, knockdown of circ_0000338 elevates the chemoresistance of CRC cells, providing a novel target for reduce susceptibility in chemo-sensitive cells [161]. Moreover, exo-circ_0005963 in CRC also contributes to improve chemotherapy with similar mechanism [55]. Those evidences reply that exo-circRNAs related chemo-sensitive or resistant can regulate the chemotherapy during the tumorigenesis and development of malignant tumors. Hence, future researches should put emphasis on exo-circRNAs to design specific tumor targeted therapy drugs. Exo-circRNAs may possibly ameliorate the awkward situation and shed new light on the of chemo-resistant patients.

### 4.5. Treating targets

In addition, the discrepancy of expression of exo-circRNAs in malignant tumors provide a penetration of tumor therapy. Emerging studies indicates that exo-circRNAs associate with several clinicopathologic features such as tumor size, lymphatic metastasis and cancer stage, which may be attenuated by artificial modification in mice. Upregulated circ-133 regulated the membrane distribution of E-cadherin and gradually enriched in the plasma exosomes as the HCC progression, promoting HCC metastasis *via* suppressing miR-133a. Interestingly, mice with higher exo-circ-133 got lower survival time and knockdown of exo-circ-133 could weaken tumor metastasis in animal experiment, which suggested its potential therapeutic ability [162]. Furthermore, circEPB41L2 was downregulated in the CRC exosomes, which stimulated CRC cell proliferation but decreases apoptosis by suppressing miR-942-5p and improving the and PTEN/AKT signal pathway *in vitro*, while overexpression exo-circEPB41L2 certainly weakened tumor growth and reversely enhanced tumor growth *in vivo* [163]. Other higher exo-circRNAs such as exo-circFBLIM1 [164] in HCC, exo-circRanGAP1[53], exo-circNRIP1 in GC[52], exo-circLONP2 [54], exo-circ-ABCC1 [56], exo-circRHOBTB3 [165] in CRC, exo-circ-PDE8A, exo-circ-IARS [26,56] in PDAC also have the potential to become new target for tumor therapy.

Besides, those down expression exo-circRNAs also present similar function. Exo-circ-005143 is an antioncogene that significantly downregulated in HCC tissue *via* acting as a sponge for miR-331-3p and increasing the expression of BAK1, which arrests the cell cycle in the G_0_/G_1_ phase, furtherly promotes the tumor growth. *In vivo*, overexpression exo-circ-005143 can remarkably reduce tumor volume and weight, suggesting its potential value for clinical application [84]. Owing to their unique structures and wide impacts, circRNAs may make a profound impact in designing targeting strategies in cancer.

Nowadays, researchers have found that exo-circRNAs can be transported to immune cells as tumor antigens to activate anti-tumor immunity, or bind to miRNAs and proteins to regulate immune cells activity. In addition, when exo-circRNAs are transported from tumor cells to immune cells, they can silence the relevant target genes by helping release miRNAs into immune cells [166]. For example, exo-circRNA from adipocytes promotes the growth of hepatocellular carcinoma by targeting deubiquitination associated USP7 [167]. It was reported in 2016 that circRNAs could be downregulated and transferred to exosomes from KRAS mutant colon cancer cells [168]. The function and potential applications of exo-circRNAs in TME deserve recognition and may be gradually revealed in future studies.

## 5. Conclusion and future prospects

The nascent era of the application of exosome and its cargo is gradually exploding. Though, there are several limits about exo-circRNAs. Firstly, it is urgent to establish a uniform naming convention to unify the database of exo-circRNAs. For instance, cirs-7 indicates that CDR1, related to the CDR1 gene, acts as a sponge for miR-7, both referring to a same circRNA [23]. Secondly, current databases exist repeating and duplication, which can’t contact with each other and cause the waste of resources. Then, the costs of purifying exo-circRNAs are still expensive indeed and the use of exo-circRNAs as biomarkers or treating targets in clinic are still remains years away. Fourth, the characteristics and mechanisms of exosomes and exo-circRNAs and exosomes are not only here [169]. Cell biologists developing techniques should also focus on the optimized isolation procedures to extract special exosomes at the single-vesicle scale and circRNAs that may associate with special disease. The initiation and progression of cancer associated with multi-factors, multi-steps, multistage, so to rely on only single exo-circRNA for diagnosis and treatment isn’t specific to clinical utility. Last but not the least, current researches are still theoretical and inadequate so that we should increase the funds and energy in translational medicine and transformation studies of exo-circRNAs. Further investigations are required to establish new models *in vivo* combined with powerful imaging methods and fully resolve the problem of transforming from bench to the beside.

This review of exo-circRNAs’ biogenesis, mechanisms in digestive malignant tumors and applications provide novel theories and methods, meanwhile, mounting evidences presented within this overview exhibit their potential value. Loaded with diagnosis and therapeutic contents, we believe exosomal circular RNA will ultimately be engineered and come into service for highly selective and effective services in all kinds of diseases.

## Supplementary Material

Table S1.docx

## Data Availability

Data sharing is not applicable to this article as no new data were created or analyzed in this study.
