# Supplementary material for: Biological functions and molecular mechanisms of exosome-derived circular RNAs and their clinical implications in digestive malignancies: the vintage in the bottle
Source: Ann Med. 2024 Nov 1;56(1):2420861. doi: 10.1080/07853890.2024.2420861 (PMC11536637; doi:10.1080/07853890.2024.2420861)
Supplement: Table S1.docx [file IANN_A_2420861_SM2628.docx]

Supplementary Table 1 Diagnostic and prognostic exo-circRNAs

| Marker type | Exo-circRNA | AUC | specificity | sensitivity | HR  values |
| --- | --- | --- | --- | --- | --- |
| Diagnostic markers | exo-circ-0004771 | 0.816 | 80% | 81.43% | - |
|  | exo-circ-PNN | 0.854 | 69.0% | 91.7% | - |
|  | exo-circ_0065149 | 0.640 | 90.2% | 48.7% | - |
|  | exo-circ-KIAA1244 | 0.7481 | 68.00% | 77.42% | - |
|  | exo- circ50547 | 0.900 | - | - | - |
|  | exo-circ_0043603 | 0.836 | 92% | 64% | - |
|  | exo-circ_0004001 | 0.79 | 81.25% | 76.19% | - |
|  | exo-circ_0004123 | 0.73 | 84.38% | 66.67% | - |
|  | exo-circ_0075792 | 0.76 | 68.75% | 80.95% | - |
|  | exo-circ_0028861 | 0.79 | 82.69% | 67.86% | - |
|  | exo-circLPAR1 | 0.858 | - | - | 0.46 |
| Prognostic markers | exo-circRanGAP1 | 0.646 | - | - | 1.43 |
|  | exo-circ-PDE8A | - | - | - | 1.764 |
|  | exo-circ-0000419 | 0.642 | 67.7 % | 55.2 % | - |
|  | CircTMEM45A | 0.888 | - | - | - |
|  | exo-circCOG2 | 0.675 | - | - | 2.70 |
|  | exo-circPDK1 | - | - | - | 2.172 |
|  | exo-circ_0026611 | 0.724 | 0.529 | 0.800 | 3.79 |
|  |  |  |  |  |  |
